# Budget Process and Execution: A Case Study on the Underperformance of the Peruvian Health System, 2000–2021

**DOI:** 10.9745/GHSP-D-23-00250

**Published:** 2024-04-29

**Authors:** Rolf Erik Hönger, Doreen Montag

**Affiliations:** a Wolfson Institute of Population Health, Queen Mary University of London, London, United Kingdom.

## Abstract

**Introduction:**

Health system financing for emerging economies with aging populations and changing epidemiological profiles is an increasing challenge. Peru, as one of the countries with the highest economic growth in this century, provides a good example for analyzing the impact of the budgeting process and the budget execution on the performance of a health system. This article aims to answer how policies and processes are the root causes of the performance gap of the Peruvian health system.

**Methods:**

We used a case study methodology composed of 17 semistructured interviews with senior national and regional actors conducted between the end of 2021 and the beginning of 2022. Participants were selected with a combination of purposive, convenience, and snowball sampling until reaching saturation at 14 interviews. Participants’ answers were grouped according to the topics explored, enabling comparisons and identification of themes.

**Results:**

The responses revealed that 4 interconnected influences affect the Peruvian health system. Political instability affects the sustainable development of health policies. The fiscal cycle limits the public health expenditure to a low 3% of the gross domestic product. The budget cycle is based on the low motivation of the Ministry of Health (MOH) to establish a proper budgeting process. The execution cycle represents the results of chronic underinvestment with a lack of professionals, equipment, and data affecting the access to care expressed by a high out-of-pocket share in health expenditure.

**Conclusion:**

To escape these cycles, the MOH needs to be able to argue in economic terms for the prioritization of health, showing the economic rationale for investment in health. Taxes need to finance the additional investment within the available fiscal space. The rigidity of the budget law needs to be adapted, and a technical budget that is oriented to the current and future health priorities needs to be elaborated.

## Abstract (precis)

Four interconnected influences have caused the Peruvian health system to underperform. To break the influence of these cycles, the Ministry of Health needs to argue in economic terms to prioritize health.

## Teaser

**Key Findings:**

- Although the recent economic growth of Peru has enabled the country to lift a large portion of its population out of poverty and improve health indicators, the health system is still fragmented and underfinanced. The population has a high out-of-pocket share and difficulty accessing health services, with many lacking insurance coverage.
- Interviews with former ministers of health and other senior officials revealed that 4 interconnected influences have negatively affected the Peruvian health system: (1) Political instability and frequent changes in health leadership at the ministry and regional levels have resulted in inefficiencies and restrictive budgeting laws. (2) This has, in turn, resulted in low investment in health and limited fiscal space. (3) This restrictive budget, combined with lack of planning and management capabilities, results in low-quality health expenditure and low budget execution. (4) Together, these factors lead to a lack of human resources, data, and technology that hinder access to health care and fuel high out-of-pocket spending.

**Key Implications:**

- Strong leadership and political support are needed to argue for the economic benefits of investing in and prioritizing health.
- Data infrastructure should be strengthened to help with decision-making for budget planning, execution, human resources, and changes in populations’ health needs.

## INTRODUCTION

Health system financing in the pursuit of universal health coverage (UHC) is facing difficulties in many countries as populations age and health care becomes more expensive.^1^ Especially in emerging markets, countries with rapid economic growth need to direct increasing funds toward social development for populations that have increased expectations.

Between 2000 and 2020, Peru had the second biggest economic growth in Latin America and the Caribbean, after Panama, achieving upper-middle-income status.^2^^,^^3^ This economic growth allowed the improvement of the health system and an important advance toward UHC.^4^ Poverty fell from 58% to 23% of the total population. Stunting of children aged younger than 5 years was halved to 14% in 2015.^2^^,^^5^ Important health indicators like maternal mortality (265 per 100,000 live births in 1990 reduced to 68 in 2015) and infant mortality (56 per 1,000 live births in 1990 reduced to 10 in 2020) improved significantly.^6^ The prevalence of diseases, such as malaria, TB, and other tropical diseases, that affect marginalized populations decreased.^4^^,^^7^

Although Peru experienced impressive progress, it still lags behind its Latin American peers.^8^ The health system performs more like a lower-middle-income country.^9^ Velazquez et al.^10^ summarized the health system in Peru as fragmented, segmented, and underfinanced and has a high out-of-pocket (OOP) share, health care that is difficult to access, human resources that are unevenly distributed and with low salaries, and lack of health insurance coverage for many, especially the poor.

The preparedness of the health system in Peru during the COVID-19 pandemic was especially poor, with Peru having the highest excess mortality per million inhabitants.^11^

## CHARACTERIZATION OF THE PERUVIAN HEALTH SYSTEM

Due to economic growth over the 20 years before the COVID-19 pandemic, Peru has dramatically improved its living standards and basic health indicators.^2^^,^^5^ UHC has been a goal since the 2009 and 2013 health reforms, strengthening governance and oversight of the health system and increasing insurance coverage,^12^^,^^13^ accelerating to 96% insurance coverage during the pandemic.^6^ However, a high OOP expenditure of 29% reflects that insurance coverage does not mean adequate access to health.

The Peruvian health system is characterized by its fragmentation into different vertically integrated health systems. Because of the decentralization of government competencies in 2002, an additional 26 regional health organizations were added.^4^^,^^5^^,^^8^^,^^14^^,^^15^ There are 4 principal subsystems for financing and service provision: the tax-financed public sector (SIS), the contributive sector (ESSALUD), the smaller subsystem of the armed forces and police, and the private sector with its health insurance companies and private clinics. All of these subsystems cover different subsets of the population with different benefit packages and with their independent health providers,^8^^,^^15^^,^^16^ leading to a system that has a poor adaptation capacity to changes in demographics and epidemiology.^5^^,^^17^ The separation of the SIS from ESSALUD leads to duplication of efforts because of a lack of shared learnings, investments, and resources (less than 1.3% of the SIS budget purchases services from the better-equipped ESSALUD).^18^ It also increases the risk of a resource allocation that is not in the national interest,^8^^,^^15^ especially as both networks are complementary.^16^^,^^17^^,^^19^^–^^21^

The Peruvian health system is fragmented into subsystems for different subsets of the population, leading to a system that has a poor capacity to adapt to changes in demographics and epidemiology.

The lack of well-prepared decentralization is a widely recognized issue affecting the performance of the Peruvian health system.^5^^,^^8^^,^^14^^,^^22^^,^^23^ The regional governments do not have sufficient capacity and capabilities to plan, coordinate, and deliver services, nor to implement national strategies.

Apart from these systemic issues, the literature recognizes the lack of sufficient financing as a fundamental issue.^24^^,^^25^ Peru invests about 5.5% of its gross domestic product (GDP) in health, which is below the 8.9% average of Organisation for Economic Co-operation and Development (OECD) members and less than the 7.2% average of its peers in Latin America.^4^^,^^9^^,^^16^^,^^21^^,^^22^ The share of public resources in health is 58.7% compared to 73% in OECD members.^5^ It has one of the lowest expenditures per GDP in the region, just above Venezuela and Paraguay.^11^

Peru lacks data systems, leading to inadequate decision-making and budgeting.^5^^,^^6^^,^^20^^,^^22^ Rajan et al.^26^ and White^27^ identified the budgeting process as a key factor in the health system defining the allocation of resources. Budgets tend to be historically based that do not align with current and future health needs.^22^^,^^28^ There are no incentives for improving health outcomes or the efficiency of expenditure because payments are based on production.^2^^,^^16^^,^^21^^,^^22^ The budget allocation is still struggling to prioritize non-communicable diseases that currently account for 66% of the mortality and only 3.2% of the total public health budget allotted to it.^29^ In particular, the SIS is underfunded, as allocations are based on historic levels and not per capita in response to the expansion of the insured population.^5^^,^^6^

The World Health Organization handbook^26^ and the Inter-American Development Bank^30^ express that the concept of budgeting for results was created to increase the efficacy and efficiency of health expenditure. Successful implementation of this process requires improved administrative capacity, high-quality information, political support for prioritization, and clearly defined incentives for target fulfillment.

In Peru, only since 2008 has a law regarding the budgeting for results concept existed.^31^ Only 7 vertical programs were implemented in health, representing about 50% of the public health expenditure.^6^^,^^15^ Positive examples of budgeting for results have been reported in HIV and malnutrition, with mixed results in other areas.^2^^,^^32^^,^^33^ While recognizing the results of these vertical programs, their application has been widely criticized by researchers of the OECD and the World Bank as not focusing on a systemic health impact but using 50% of the total budget.^2^^,^^5^^,^^6^^,^^14^ Even promoters of results-based budgeting, like the OECD and others,^8^^,^^14^^,^^22^ recognize that the process of budgeting individual line items is too rigid and does not allow the required flexibilities for improved outcomes. As results-based budgets do not have the intended effect when there are no consequences of bad management and lack of capabilities, technical experience, and good data, the budgeting for results had limited impact in Peru.^22^

For 2020, the national health budget execution in Peru was 88% of the allocated amount, but 86% of the allocated amount for the central government, 91% for regional, and only 66% of the amount for municipalities was executed, leaving 3.4 billion of soles (approx. US$1 billion) unexecuted.^34^

Montenegro-Idrogo and Chiappe^35^ showed the devastating impact of low budget execution, as there was a clear negative relationship between budget execution and COVID-19 deaths in the first months of the pandemic in their analysis of the impact of the regional budget execution in Peru.

This case study collected interview responses from health leaders of different levels and ministries in an effort to understand what affects the Peruvian health system’s performance. We argue that the budget process and the lack of budget execution are part of a vicious cycle that reinforces the lack of resources, lack of investment in trained human resources, and lack of infrastructure and data, hindering improvements in the process and the execution. Under tight fiscal management, the lack of prioritization of health in the pursuit of UHC is a key challenge when health is not considered an investment but rather an expense. This fiscal primacy combined with the country’s political instability during recent years explains the underinvestment and underperformance of the Peruvian health system against its peers and offers valuable lessons for other emerging markets on what to avoid to build a strong system that can guarantee UHC.

## METHODS

To analyze the complexity of this phenomenon, we used a qualitative case study, which can be criticized for the risk of not being generalizable but has the strength of providing a holistic view of phenomena.^36^^,^^37^ By carefully choosing the participants of the study, the result can be claimed as being representative. A qualitative case study facilitates the exploration of an issue within the respective context using different data sources, allowing diverse views to detect the variations of the phenomena coming to the surface.^12^^,^^38^ The qualitative case study is a constructivist paradigm, claiming that the truth is subjective while letting the participants tell their version of the story. Therefore, this methodology allows for studying in-depth, real-life phenomena.^37^ As Yin^39^ outlines, case studies should be used when the focus is how and why questions, behavior manipulation is impossible, there is a need to cover contextual conditions, and clear boundaries between context and issue exist. As this article analyzes how and why the budget process affects the health system through the lens of leaders with budget influence within the actual political conditions, all 4 elements of Yin are included.

An explanatory single case study with embedded subunits was selected to detect potential causal links between the budgeting process and budget execution with the performance of the Peruvian health system.^12^^,^^13^ The subunits are the different levels of the health system, considering ministerial levels, including the Ministry of Economy and Finance (MEF) and regional health director views.

### Semistructured Interviews

We interviewed former or current health leaders at different levels of the health system and responsible persons at the MEF for the health budget.^40^ The interviews aimed to find patterns in general and on the different levels of the system to verify the hypothesis of the importance of the budgeting process and elaborate policy recommendations to remedy the detected issues.

The development of the interview guide was based on the core topics of the research question to understand the role of the budget process in the underperformance of the Peruvian health system.^41^^,^^42^ The interview was semistructured with an interview guide, allowing sufficient adaptations depending on the participant’s expertise and willingness. REH conducted all interviews and was able to follow more in-depth leads mentioned by the participants to gain deeper insights into their expertise.^37^^,^^42^

After conducting the first 2 interviews, the interview guide was adapted to get a better flow of the conversation.^42^ The interview transcript and insights were shared with the participants to ensure the research reflected their experiences and insights.

### Sampling

The sampling strategy needed to allow insights into different levels of the health system and to include the MEF, as they assign the budgets to the system and define the process. To guarantee that the information provided by the participants was accurate, we interviewed several participants of each subunit. To select the participants from each subunit, the following criteria were used for stratification^43^^,^^44^:

- Ministers of health: last 12 years, long enough tenure (more than 3 months), and previous functions in the health ministry or regional director positions, leadership
- Vice ministers: last 12 years, responsible for budget or important programs
- Regional health directors: last 12 years, long enough tenure (more than 6 months), ensure representation of the 3 Peruvian regions (Coast, Andes, and Amazonas)
- Health ministry advisors: last 12 years, recommended by others as relevant in the budget process or profound knowledge of the political process
- MEF: responsibility for the health budget in the last 12 years

Using these criteria of purposive sampling, the first group of health leaders was identified and contacted.^36^^,^^43^^,^^44^ The ministers were relatively easy to identify, but participants in other subunits were not. To start the process, the first author used convenience sampling by calling upon known ex-ministers and regional health leaders.^36^^,^^43^ By asking the participants whom they would recommend as experts in the research question according to the predefined subunits, we used snowballing combined with purposive sampling, as the recommended persons needed to fit the selection criteria.^36^^,^^43^^–^^45^ To minimize bias, we recruited participants whose names were mentioned several times, especially for the subunits of vice ministers, regional health directors, advisors, and the MEF.

The sample size is not defined in theory until there is a point of data saturation when the participants do not come up with new relevant information.^36^^,^^38^^,^^43^ Thematic and meaning saturation was reached at 14 interviews, and recruitment stopped after 17 interviews.

### Data Collection

Before the interview was conducted, participants gave their written consent. Interviews were conducted in Spanish for 1–2 hours, depending on the willingness of the participant to deepen the conversation. All were recorded and transcribed to ensure appropriate representation of their thoughts and to reduce the risk of loss of data. Recordings, transcripts, and the draft article were shared with participants.

### Data Analysis

For the interview analysis, participants were anonymized into subunits mentioned, while combining vice ministers and advisors to have a minimum quantity per segment. The transcripts were used to identify key themes that related to the research question according to each subunit.^38^ Answers were grouped per identified key topics based on the questions. The questionnaire proved to be well structured, facilitating the analysis. Organizing the questions in columns for each participant in Excel allowed for comparisons of answers and identification of themes. Insights were derived by outlining commonalities and differences between interview groups and pointed to individual experiences and opinions.

### Ethical Approval

Before interviewing, the investigation received written approval from the ethics commission at the Queen Mary University of London with the number QMERC20.584.

## RESULTS

We interviewed 4 former health ministers, 6 former vice ministers and senior advisors, 3 regional health directors, and 4 senior officials from the MEF. The sample includes 4 women, but insights did not differ by sex but rather by the respective experience in the health system.

### Interconnected Influences Negatively Affect the Health System in Peru

**FIGURE 1:**
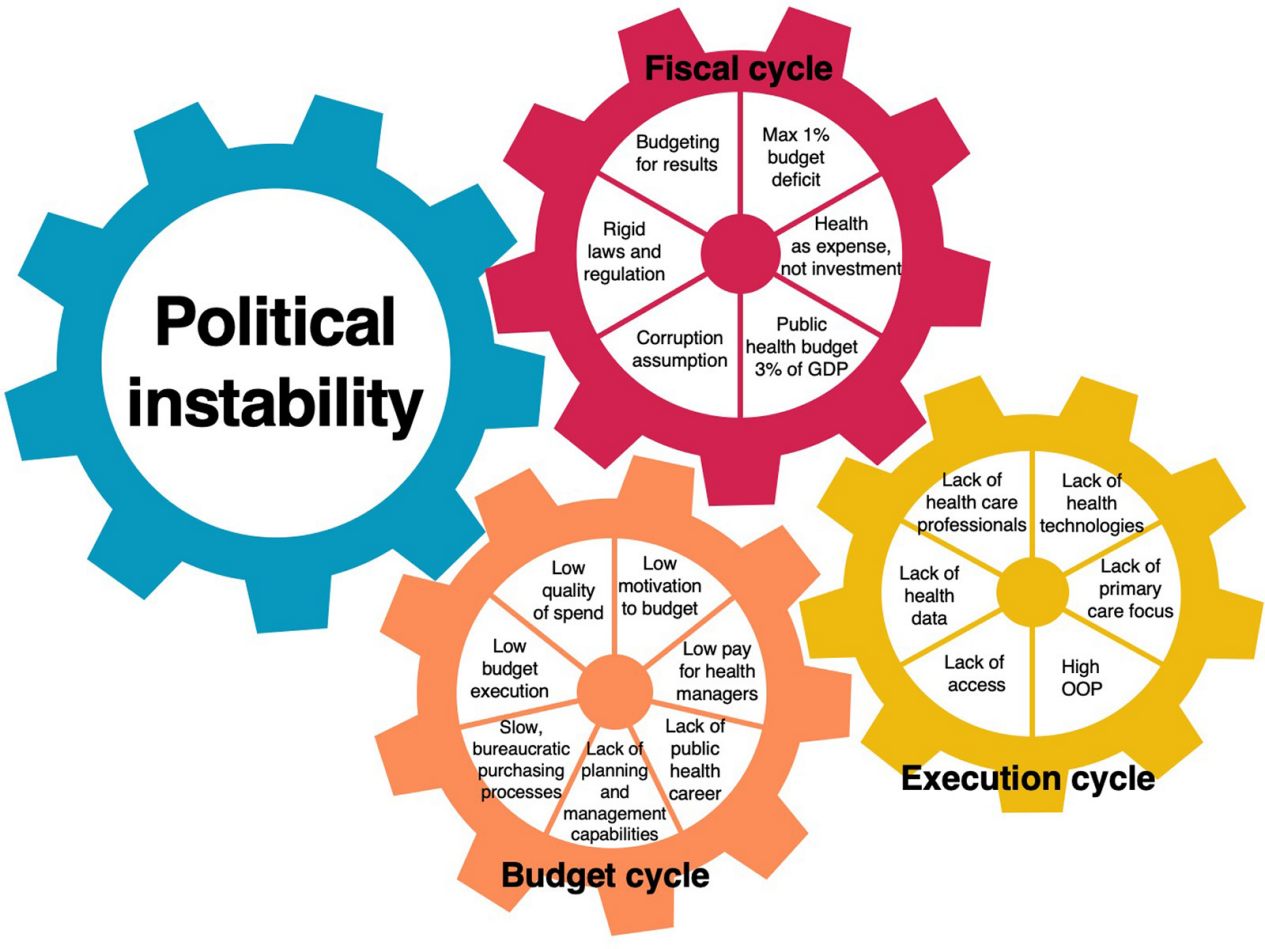
Interconnected Influences Influencing the Peruvian Health System Abbreviations: GDP, gross domestic product; OOP, out-of-pocket.

The participants’ responses brought to light the interdependencies of 4 interconnected influences that severely affect the Peruvian health system (Figure 1): political instability, fiscal cycle, budget process cycle, and execution cycle. The main influence was political instability, which together with the fiscal cycle that is based on the fiscal primacy of the government, are the key drivers of the process. The budget process cycle is conditioned by the fiscal cycle and influenced by political instability, but the necessary reforms could, in turn, influence the fiscal cycle. The execution cycle represents the consequences of the current conditions, but the right investment and policy decisions could influence both the budget and fiscal cycles.

Participants’ responses highlighted that political instability and fiscal primacy are the key factors influencing the health system performance.

#### Political Instability

Several participants mentioned the negative influence on the health system due to the political instability and constant changes in the health leaders—reflected in 14 health ministers and 35 vice ministers during the last 10 years. The same instability of health leaders existed at the regional level, as all regional health directors mentioned. A former vice minister mentioned that this lack of continuity “makes politics much more important than any health policy and the appetite for fundamental changes is little.”

#### Fiscal Cycle

**FIGURE 2:**
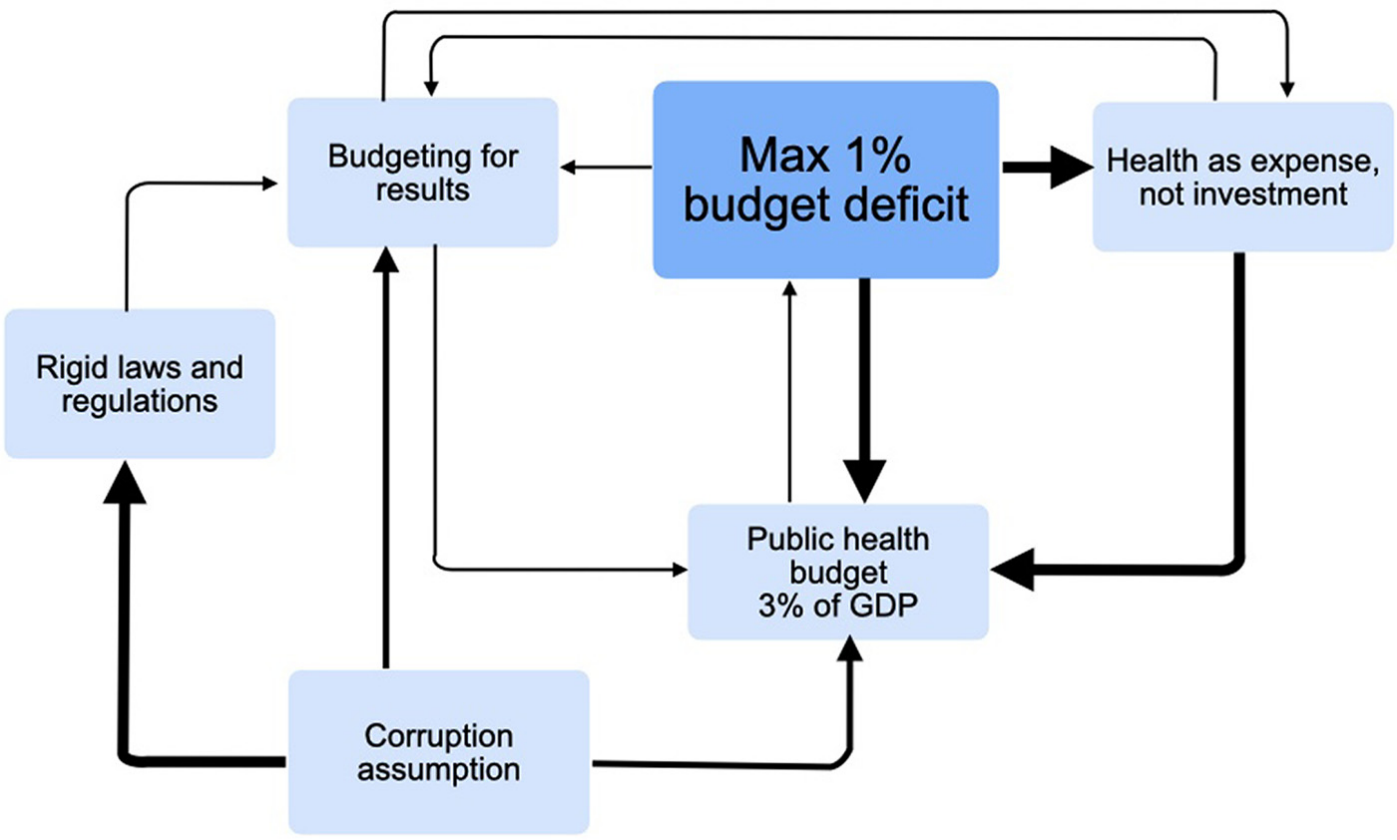
Relationships in the Fiscal Cycle Abbreviation: GDP, gross domestic product.

The fiscal cycle (Figure 2) is the dominant cycle and highly influenced by political instability. Considering the overall objective of the Peruvian government has been to avoid a fallback into the crises of the 1980s, the fiscal primacy of a maximum of 1% of the budget deficit has been maintained since the beginning of the 1990s. As health has been viewed as an expense and not an investment, public health has not been prioritized within the available budget. With the general assumption that there is abundant corruption, the laws regulating budgets have been extremely rigid as well in the budgeting for results programs. The fiscal cycle cements the low investment in health.

*The total fiscal income of Peru is 20% of GDP (average Latam above 26%, OECD above 40%) out of which the tax revenue is only 14%. This defines the capacity of financing. Moreover, with the fiscally responsible management that distinguishes Peru, it limits the fiscal space. Therefore, the increase in financing depends on the economic growth and the degree of formality in the Peruvian economy*. —MEF representative

This was well recognized by many individuals in the MOH and regional health directors. Another MEF representative added that the law allowed a maximum budget deficit of 1%, making the finance minister directly accountable and limiting the willingness to experiment.

Most respondents agreed that the 3% of GDP invested in public health was too low. One vice minister added that with the lack of fiscal space and the lack of execution of the current resources, there was little pressure to assign more resources to health.

*Today’s agenda is about what to do and use better the existing resources*. —Former health minister

*The MEF sees health as a cost and not as an investment.* —Former vice minister

*There are a lot of signals that the spending is inefficient and affected by corruption. Therefore, the MEF insists on rigidity. There is no trust in the MOH.* —MEF representative

The resulting rigidity was severely criticized by the MOH representatives at all levels.

*We need to change the law of budget, as this law is the mother of the rigidness of the budget.* —Former health minister

*The control organ sees an error or deviation and immediately signals corruption, even if there is a real error. This leads, together with the lack of continuity, the lack of proper training and lack of capabilities, to paralysis.* —Former health minister

A former health minister and a former vice minister explained that purchasing processes need approval to go through 8–20 persons and commissions, most of whom have little or no technical knowledge.

Moreover, an MEF representative and a former vice minister explained that the fear of committing an error in purchasing was very high because even presidents were in jail for corruption. A former vice minister expressed that the “terror of corruption is above the needs of the patients.”

MOH and MEF representatives agreed that many norms needed to be abolished or improved. Moreover, DR1 adds:

*Incentives are wrong as you are measured on whether you spent the budget, but not if the intervention makes sense and the expense was of quality.* —Regional health director

Budgeting for results was mentioned as being introduced as a management tool for prioritized health situations in an extremely rigid form, not allowing a change from 1 budget to another nor between line items. A former health minister described the budgeting for results process as chaotic and very complicated due to the rigidity and lack of adaptation to the epidemiological needs of the country, as reflected in 40% of resources going to maternity and childcare despite the significant change in epidemiological profile. However, a MEF representative argued, “it is the task of the MOH to come with well-argued and documented plans to change or introduce another budget.

The role of the MEF is viewed differently by MEF representatives and different levels of the health system. Two MEF representatives viewed the MEF as the guarantor of reasonable prioritization and a barrier against corruption.

Another MEF representative explained that the MEF was the most powerful ministry in the government and that the economic crises of the past have taught that sustainability depends on fiscal responsibility, so the MEF was putting fiscal objectives above health objectives.

*If health is a priority, then you need a clearly defined program with prioritized outcomes. The president, the prime minister, the minister of health and the congress need to support to get a dedicated budget to solve the health issue as existed during Humala’s presidency. If the MEF is on your side it is much easier to reform.* —MEF representative

However, most leaders in the MOH viewed the MEF as intransigent and a barrier to public health as rules are too narrowly focused.

Concisely, the fiscal cycle represents the fiscal primacy and nonprioritization of health in the budget combined with the rigidness due to corruption assumptions. This fiscal cycle highly influences the budget cycle, as it defines the environment of budgeting and determines the conditions for the low execution of health budgets, hence reinforcing the lack of pressure to increase the health budget.

#### Budget Process Cycle

**FIGURE 3:**
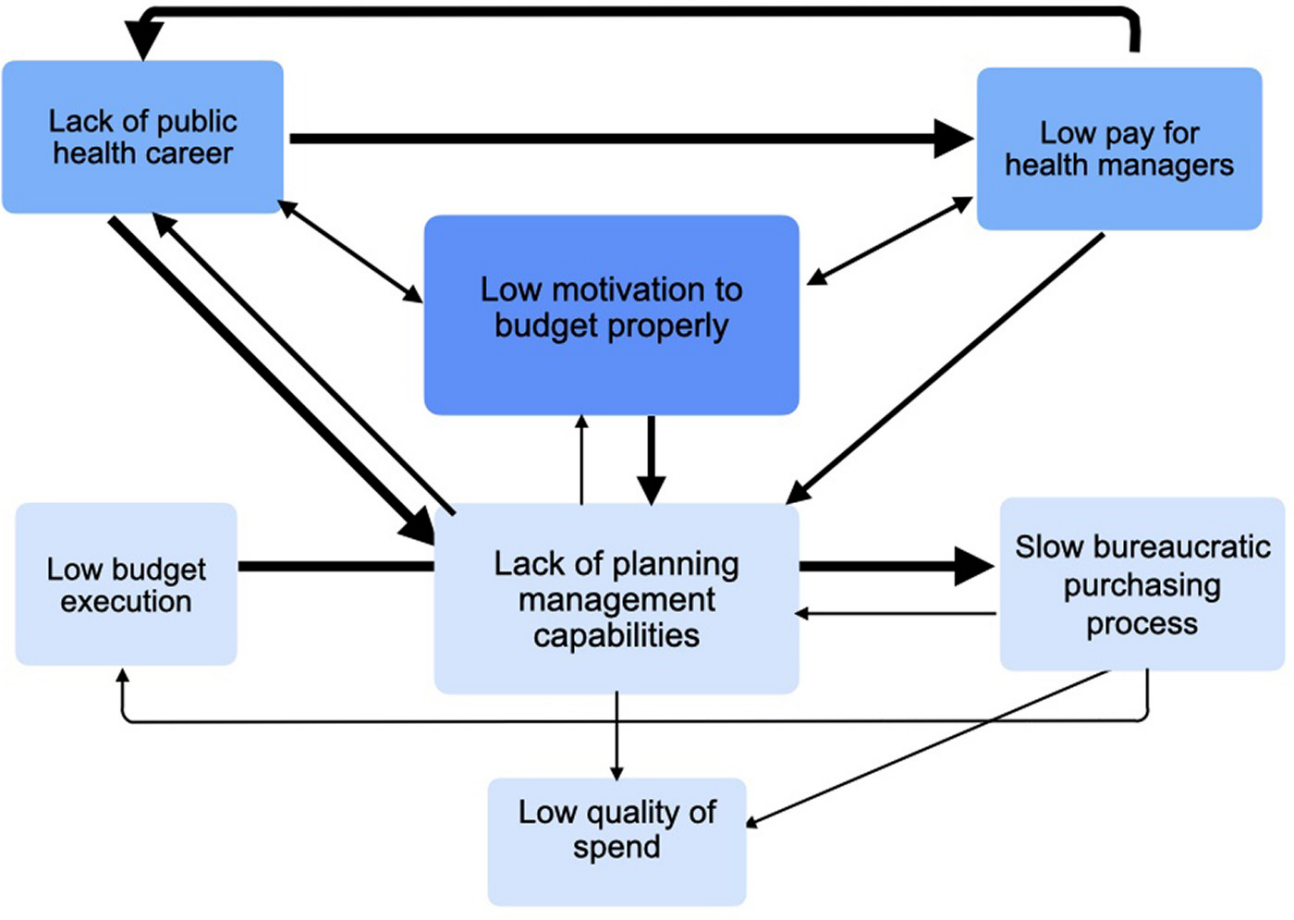
Relationships Within the Budget Cycle

The budget cycle (Figure 3) is defined by the perceived lack of sense in dedicating time to prepare and argue for a budget considering the limited fiscal space, as 2 vice ministers described. This, together with the low salaries and lack of career opportunities for health managers, leads to a lack of planning and management capabilities. Combined with the rigidness of the budgets and purchasing processes, the result is low quality of expenditure and low budget execution.

Considering the limited fiscal space, participants mentioned the lack of incentive to prepare and argue for a sound budget according to priorities.

The MEF representatives argued that the MOH did not plan well and did not defend the plans with data. However, other participants countered that even with good arguments, they hardly got resources, so there was very little incentive to invest in good planning. The most experienced health managers who could cite examples where budgets had dramatically increased because of good planning and reporting of results at the regional or hospital level did not confirm this.

An MEF representative who saw the budget as a political and not a technical instrument confirmed the lack of incentive to prepare and argue for a health budget. Another MEF representative agreed.

*The council of ministers decides on the percentage distribution of the available resources to the respective ministries and then the minister decides what the distribution in his area is. There is no technical discussion.* —MEF representative

*Why would you do all the elaboration of a plan when political pressure is much more effective?* —MEF representative

Two MEF representatives confirmed what most of the MOH leaders mentioned that the MEF defines the maximum budget based on the past and keeps a margin for negotiation.

*The MOH does not do budgeting for the needs; it adapts to the maximum amount given and as the MEF makes very pessimistic assumptions that usually are surpassed, this maximum amount does not represent the real capacity of the government expenditure.* —Former vice minister

Participants mentioned that the consequence is that from the start of the year, there are not enough resources and later assignations arrive when the slow purchasing process does not allow for timely execution.

*In theory, the region should organize a seminar to elaborate the budget, but you do not have a technical office that understands the process of how an intervention impacts a disease. There are no budget specialists that understand enough about health to elaborate a good health intervention plan. In addition, there is no sanitary intelligence enabling decision-making. Therefore, tables are filled with numbers of last year instead of a strategic process. Additionally, as you are measured whether you spend what you budgeted, and not if the intervention has the desired impact, the system reinforces that it is not necessary to do proper planning.* —Regional health director

The leaders of the MOH see this deficit of well-formed health managers caused by the absence of sustained formation of public health leadership.

For some former ministers and vice ministers, this is a consequence of the failed decentralization. Another former minister disagreed, saying that by sending specialists in planning and budgeting to the regions, a better adaptation to the needs of the regions can be achieved and decentralization made to work. However, an MEF representative cautioned, “some of the submitted budget requests read like primary school work,” resulting in many requests that did not fulfill the minimum legal and technical requirements for approval, as mentioned also by other MEF representatives.

A regional health director mentioned that a public health career is not valued.

*There is little incentive to study public health if the appointments go to the family members and friends of the governor or minister of health.* —Regional health director

Participants mentioned that there is no meritocracy and no bonus based on results. Moreover, the most competent leave for the better-paid positions in the private sector or ESSALUD, especially because the monthly salaries are very low (US$420 in the provinces vs. US$1420 for a medical doctor).

One former vice minister asked, “Why would you assume such a responsibility of managing the large purchasing processes and the risks associated with them?” Moreover, he added those who were competent went to the nation’s capital, as their salaries were 10 times higher.

*The consequence in the regions is a lot of ignorance of the norms that regulate budgets in the system, resulting in rejected proposals and delayed execution.* —Former vice minister

Quality of spending was a recognized problem. An MEF representative explained the impact of fragmentation on the quality of spending.

*The National Oncology Institute (INEN) attends 2,000 tomographic exams per month, but other hospitals with the same equipment only do 60 exams. Sometimes there are even several pieces of equipment per city in the different health systems. This waste is the result of excessive fragmentation and segmentation of the health system.* —MEF representative

Poor planning was also responsible because many adjustments were required, delaying execution in the first months of the year, as a former minister and vice minister explained. Therefore, the budget execution improved from August onward, as a former minister expressed. An MEF representative stated that about 5% of the total budget went to local governments, mainly for investment, but they had very little capacity to execute. In general, participants mentioned that budget execution was about 95% of the payroll, while the investment in budget execution was regularly only 50% due to the low planning capability of investments in the regions and municipalities.

In summary, the vicious cycle starts with no motivation to elaborate on technical budgets as the fiscal rules dominate. Public health professionals lack good incentives and career opportunities, and the available personnel are poorly qualified. The low budget execution caused by bad planning and unqualified personnel supports the MEF’s argument not to invest more in health. This is the connection to the fiscal cycle.

#### Execution Cycle

**FIGURE 4:**
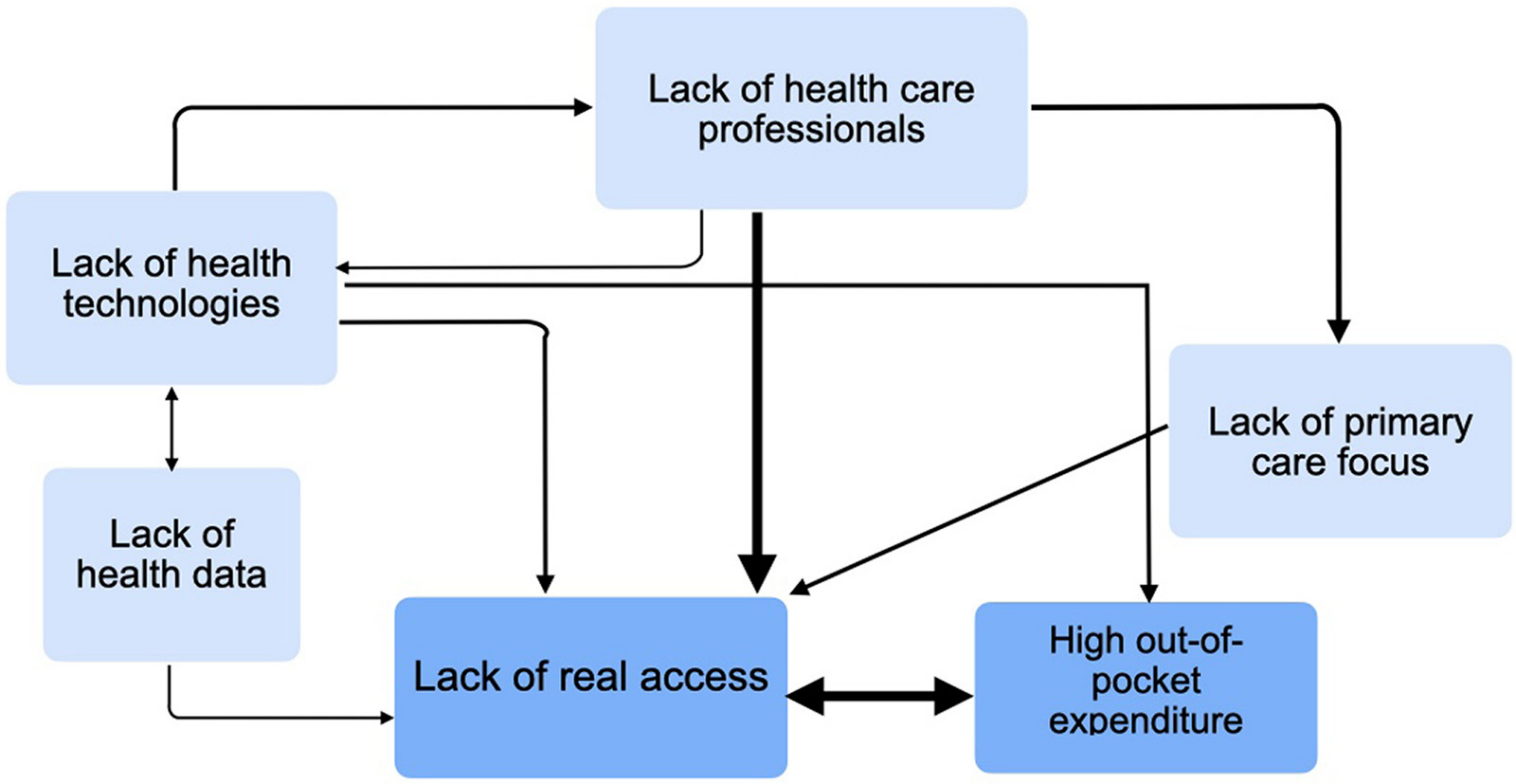
Relationships in the Execution Cycle

The fiscal and budget process cycles and political instability affect the health system by creating a lack of human resources, data, and technologies, which, in turn, result in a lack of access to health and high OOP spending. The execution cycle (Figure 4) represents the results of the fiscal and budget cycles. However, if this cycle were improved, it could have a positive effect on the capabilities of the system.

A commonly recognized issue by the health leaders was the lack of health care professionals and lack of technologies in the public setting, a direct consequence of the chronic underinvestment in public health.

A former minister explained that the consequence of not investing in data generation was, “there are no management indicators, therefore, the goals are not achieved and the plans or programming cannot be evaluated.” Moreover, an MEF representative added, “the lack of data causes as well that we do not know the quality of the expenses in health.”

*There is no decision-making based on data and evidence. There is no way to unify the [electronic medical records] to achieve actualized information, as this was never prioritized.* —Former vice minister

Representatives of the MOH, regional health directors, and the MEF agreed that, because of the low investment, there was only theoretical universal access due to availability and access issues. Both long waiting lists and lack of effective access to medicine and tests led to high OOP. The low response capacity at the primary health level caused the lack of health coverage, forcing people to seek care in overloaded tertiary-level hospitals.

*The citizens suffer from abuse, waiting list for access of 3 months and more and a centralized infrastructure that is difficult to access.*—Former vice minister

Therefore, in addition to the lack of access, the health system was perceived as not responding to people’s needs.

### Proposals for Improvement

#### Increasing Understanding of the Importance of Health Investments

COVID-19 was a great example of what could happen when the MEF believed in an issue.

*The ministry understood the importance of vaccination and it was the MEF that decided to buy vaccines, to pay extra for nurses and to make sure that all regulations were eased. It spent 7 billion soles more without a fiscal problem. If there were the same understanding for chronic diseases, the same amount could be injected into the health system.* —MEF representative

Various ministers and vice ministers shared the view that the necessary changes can only be achieved with the strong support of the president.

#### Budgeting Processes Can Be Improved With More Planning, Support, and Human Resources

A former health minister expressed that it is possible to obtain more resources with good argumentation, citing the children’s hospital showing progress against key indicators and increasing the budget from 30 million soles in 2015 to 234 million soles in 2021.

Two former health ministers and a regional health director declared that the MOH should become a policy setter and stop being a health services provider.

Many participants agreed that a well-recognized career program in public health, such as a past program called SERVIR, should be reinstalled with merit and performance-driven bonuses. A former health minister proposed that a law should define the minimum capabilities for a public health management position.

Participants agreed that a well-recognized career program in public health should be reinstalled with merit and performance-based bonuses.

Two participants argued that the control and planning in the system could be significantly improved with a good information system. For example, electronic medical records would give valuable information about which medicines in which quantities were needed in the system and allow actualized health statistics of the population for strategic decision-making.

#### All Levels of the Health System Need to Work Together

Almost all those interviewed viewed the strengthening of the primary care level as essential.

*The reforms need to make sure that the first and second levels have improved capacities to solve health issues.* —Former health minister

*This is only possible if the incentives that value hospital work much more than primary care work are broken, only possible with strong political support.* —Former health minister

*The first level needs to be able to prescribe and offer what a family needs and not be forced to refer to the next level for simple things.* —Former regional health director

Most health leaders saw the need for a unification of the health system with vertically integrated networks in the regions based on population needs. They expressed that this would eliminate the fragmentation of human resources with the standardization of salaries to avoid incentives to change from one subsystem to another and would allow better use of the installed equipment. However, this requires an injection of resources into the system. A former health minister stated that a unified system was possible if the government would pay a per capita fee to ESSALUD as a unique fund with the responsibility to manage risks and benefits for the whole population while contracting with all health networks of the different systems. Two participants expressed that to calculate the needs of a unified system, efficient cost management would be needed, but the current system does not know the cost of a particular intervention.

## DISCUSSION

Countries that, through consistent economic growth, achieve upper-middle-income status need to consider social inclusion and the improvement of their health systems to be able to respond to the changing epidemiological profile of their population. Peru is a good example of the consequences of the failure to finance an adequate health system, as demonstrated during the COVID-19 pandemic, with one of the worst performances and high excess deaths per million inhabitants.^46^

The literature about the Peruvian health system focuses on the fundamental issues of fragmentation, decentralization, and gaps in health infrastructure and recognizes the fundamental problem of the financing gap. Interviewing leaders at different levels in the health system and MEF representatives proved to be an insightful tool for learning about the impact of the current budgeting process and the lack of execution as key factors influencing the state of the Peruvian health system. The interviews highlighted that fiscal primacy and political instability are the determining factors affecting the planning and budgeting process and, subsequently, the execution of budgets.

As confirmed in a recent analysis by Gozzer et al.,^47^ participants felt that the constant change of health ministers and the lack of continuity in the management and prioritization of initiatives harmed the health system. The average tenure of a minister of health over the last 6 years was less than 6 months, while in studied 23 countries, the was about 20 months.^47^ The study reported that there was a statistically significant negative correlation between short tenures and social development indicators. Systemic improvements are hard to achieve with these short tenures due to the political instability in recent years.

### Fiscal Primacy Hampers Investments in Health

Low investment in health is central to the literature reviewed; however, there is no clear explanation of the underlying reasons.^4^^,^^16^^,^^46^ Therefore, a key insight was MEF representatives explaining that the primacy of fiscal objectives was due to the traumatic experience in the 1980s. The debt crises—caused by serious mismanagement of the government at the time—resulted in hyperinflation, external debt default, a sharp economic decline with an unsustainable debt level, a decrease in living standards, the introduction of multiple exchange rates, and the emergence of the Shining Path terrorists.^48^^,^^49^ This experience greatly marked Peruvian society and politics. Since then, a majority of the population has supported economic stability,^48^ or as Malmedier and Nagel^50^ expressed, “hyperinflation babies” want to avoid repetition of the trauma. This fiscal prudence has been maintained, even when voting for left-wing candidates, like in the last election or before with President Humala. The respective governments understood that fiscal prudence allowed growth that increased fiscal revenues for the social agenda. Due to sound economic policies, there was a significant reduction of the Gini Index in Peru driven by the emerging middle class and diminishing poverty levels, a result of the economic growth in this century.^51^

Health did not emerge as a government priority until the end of the government of Humala (2011–2016), when, in 2015, the public health investment surpassed 3% of GDP for the first time. The combination of fiscal primacy and political instability are key reasons that health has not been a priority. The leadership in the MOH is weak due to the constant rotation of the ministers. This is highly relevant to the budget process. If the MOH cannot elaborate a technically well-presented plan of why health should be considered an investment and should be prioritized, then there is little chance for political movement to provoke change. We believe that because of the strong position of the MEF within the government, the argument must be economic.

The investigators of the McKinsey Global Institute^52^ studied the economic benefit of the investment in health, concluding that emerging markets receive an economic return of between US$2 and US$4 for each US$1 invested in health. Their report, building on Edvinsson’s^53^ work on the intellectual capital of nations, argues that the main economic returns arise from better education for healthier children and the increased earning potential of a healthier population. This leads to further investment in education and production capacities. We call this the “virtuous cycle of growth” caused by investment in health. The MOH needs to argue in these terms with the MEF to achieve an increased resource allocation.

The MOH should argue for increased investment in health by describing the economic benefits of a healthier population.

We argue that to finance the increased investment in health, additional tax income would have to come from reducing the informal labor sector of Peru or increasing the tax in general or for goods affecting health like alcohol, tobacco, sugared drinks, and others. Kanavos^54^ showed that Peru has sufficient fiscal space to increase taxes earmarked for health. Matus-Lopes et al.^55^ showed the potential for a broadening personal income tax base. Kanavos and Matus-Lopes agree that the 6% target for public health expenditure can only be reached long term while addressing the informality issue of the Peruvian economy. Peru has had special taxes on alcohol (since 2013) and tobacco (since 2016), but these taxes are not earmarked for health; therefore, any increase does not automatically lead to improved budgets for health.^25^^,^^56^

### Lack of Motivation, Capabilities, and Information Hamper the Budget Process

Four main factors hamper the current budgeting process and are relevant for other countries in similar situations: (1) lack of motivation to elaborate a technical budget because having the budget does not change anything; (2) lack of capabilities due to the lack of motivation for a public health career, the mistaken notion that medical doctors were best placed to elaborate budgets, low pay, and lack of meritocracy and contributing to not being able to argue well for resources; (3) lack of up-to-date information for the leaders to make a proper diagnosis of the system; and (4) rigidity of historical budgeting that did not allow adaptations to current needs.

In this respect, the lack of governance and leadership in health mentioned in the literature are principal reasons that there are no overarching health policies. Because ministers currently change quickly, participants agreed that improvisation attending urgent matters supersedes strategic planning. Without strategic guidance, the historic inertial budgeting process repeats the past patterns, and no progress is achieved, as described well in the literature.^2^^,^^15^^,^^17^ This can be seen in the lack of leadership role of the MOH in adapting the budgeting for results process to the epidemiological profile, something that all MEF representatives clearly declared as a responsibility of the MOH and Cardenas et al.,^29^ James et al.,^15^ and Yactayo Chavez^28^ mentioned in their research. Another contributing factor is the lack of up-to-date health data for decision-making. The Inter-American Development Bank has a credit agreement with Peru to help develop the necessary digital infrastructure.^57^ Unfortunately, since it was signed in 2020, there has been little progress, as mentioned by a former vice minister.

Lack of leadership has also been reflected by the abolishment of the public health career (SERVIR) at the beginning of President Kuczynski’s government. None of the subsequent ministers have reintroduced the formation of public health specialists, as the SERVIR program had provided earlier. The proposal of a former health minister for a law that specifies the necessary requirements for health leader roles seems to be very reasonable. However, attracting capable people is only possible if the compensation policy issue is solved, as several participants mentioned. Without capable people who are trained in budgeting and have experience with decentralized health structures and the necessary political support, the elaboration of health budgets according to strategic health needs will not happen. In this respect, we believe the literature discussed more about the symptoms and less about the root causes of the issues. Poor budgeting and budget execution are not caused by decentralization per se but rather by the fiscal primacy that leads to no motivation to invest in budgeting, which does not incentivize the careers of competent public health managers. This, together with the rigidity and bureaucracy of the purchasing processes, as described in the interviews and the literature, leads to low budget execution and low quality of spending.^8^^,^^58^

Corruption is widespread.^59^^,^^60^ This corruption has led to a very restrictive budget law that does not allow flexibilities that, according to a former vice minister, exist in other countries like Chile. This is consistent with the investigations of Dale et al.^14^ and James et al.^15^ It appears that the public controllers have too much power, as the fear of prosecution makes budgeting and expenditure decisions risky.

The consequence of the nonprioritization of health can be seen in that only 28% of the citizens trust their health system.^61^ A former regional health director expressed that the concept of UHC is nice in theory but only gives people theoretical access because the offered UHC increased the demand for health services but was not accompanied by an increase in coverage.^61^ The severe lack of trust in primary care services based on the limitations of the services provided and the perception of poor quality and abuses have driven people to look for solutions either in the pharmacies or larger hospitals, especially at tertiary levels in big cities.^25^^,^^59^^,^^60^ Benites-Zapata et al.^62^ showed that, in 2015, more than 50% of the population did not use the official health institutions when they had symptoms, disease, or even accidents and usually used self-medication or the pharmacy instead of official institutions.

For other countries, the issues faced in Peru can be avoided when guaranteeing the right incentives for proper health planning, budget allocation according to priorities, long-term investment in data, technology, and the formation of health care professionals with career plans to keep them in the public system. OECD/World Bank data^24^ shows that peer countries with similar GDP/capita levels, like Colombia and Ecuador, have been more successful in avoiding the issues of Peru and giving their citizens better quality access to health.

### Limitations and Further Research

Although many of the people we interviewed had or still have executive experience in the management of hospitals, health services in municipalities, and other executive functions, our investigation did not include specifically hospital directors or the planning and budgeting directors of these institutions. Further research analyzing budgeting and budget execution at hospitals and municipalities could provide further insights.

A deeper investigation into the MEF perception of health, successful examples of health budget increases, and good execution is warranted to gain further insights into how the system can be influenced positively. An investigation of which projects or ministries achieved considerable change in allocation and the process behind it could offer additional learnings.

This study did not investigate other potential issues of the Peruvian government, such as general public investment, the government purchasing system, the judicial system, or the general budget definition of the state that goes beyond the direct health-related topics.

## CONCLUSION

To improve the Peruvian health system and offer real UHC to the population, we have shown that there needs to be a systemic improvement in health system financing and strategic planning for the health needs of the population. The research shows that understanding how a health system is financed, priorities are set, and budgets are elaborated are key for any potential intervention to improve health system performance. The existing literature is often descriptive of the issues but does not go into the identification of the root causes, as shown in this research. The budget process is a key determining factor in the performance of the health system and needs to be investigated in any work about a country’s health system.

## Notes

**Peer Reviewed**

**First published online:** March 20, 2024.
